# Associations between antimony exposure and glycated hemoglobin levels in adolescents aged 12–19 years: results from the NHANES 2013–2016

**DOI:** 10.3389/fpubh.2024.1439034

**Published:** 2024-10-17

**Authors:** Baoying Feng, Peng Tang, Sheng He, Zhenren Peng, Yan Mo, Liqiong Zhu, Qiufen Wei

**Affiliations:** ^1^Maternal and Child Health Hospital of Guangxi Zhuang Autonomous Region, Nanning, Guangxi, China; ^2^Birth Defects Research Laboratory, Birth Defects Prevention and Control Institute of Guangxi Zhuang Autonomous Region, Nanning, Guangxi, China; ^3^Guangxi Clinical Research Center for Pediatric Disease, Maternal and Child Health Hospital of Guangxi Zhuang Autonomous Region, Nanning, Guangxi, China; ^4^Department of Epidemiology, School of Public Health, Guangxi Medical University, Nanning, Guangxi, China; ^5^Birth Defects Research Laboratory, Guangxi Key Laboratory of Reproductive Health and Birth Defect Prevention, Nanning, Guangxi, China

**Keywords:** adolescents, antimony, glycated hemoglobin, National Health and Nutrition Examination Survey (NHANES), heavy metals

## Abstract

**Objective:**

This study aimed to investigate the association between antimony (Sb) exposure and glycated hemoglobin (HbA1c) levels in adolescents.

**Methods:**

A cross-sectional study of 751 adolescents aged 12–19 years was conducted via the National Health and Nutrition Examination Survey (NHANES, 2013–2016). Survey-weighted linear regression and restricted cubic spline (RCS) analyses were applied to evaluate the relationship of urinary Sb exposure with HbA1c.

**Results:**

A significant relationship was observed between urinary Sb concentrations and HbA1c levels (percent change: 0.93; 95% CI: 0.42, 1.45) after full adjustment. After converting urinary Sb levels to a categorical variable by tertiles (T1–T3), the highest quantile was associated with a significant increase in HbA1c (percent change: 1.45; 95% CI: 0.38, 2.53) compared to T1. The RCS models showed a monotonically increasing relationship of urinary Sb with HbA1c. Subgroup analyses revealed a sex-specific relationship between urinary Sb exposure and HbA1c with a significant positive association in males and a non-significant positive association in females. Sensitivity analyses further confirmed the relationship between urinary Sb and HbA1c, even after excluding participants who were overweight or obese (percent change: 1.58%, 95% CI: 0.88, 2.28) and those with serum cotinine levels ≥ 1 ng/mL (percent change: 1.14%, 95% CI: 0.49, 1.80).

**Conclusion:**

Our findings indicated that increased Sb exposure may correlate with higher HbA1c levels, especially in male adolescents. More studies are needed to further explore and validate the potential mechanisms.

## 1 Introduction

Adolescence is widely acknowledged as one of the pivotal phases in human life and is characterized by substantial changes in body composition, hormonal profiles, and metabolic functions (1, 2). These profound biological transformations have far-reaching implications for the overall health of adolescents, particularly concerning metabolic health, where glucose metabolism is assumed to be of paramount importance (3, 4). Glucose metabolism is an intricately orchestrated physiological process encompassing insulin secretion, tissue-specific glucose sensitivity, hepatic glycogen synthesis, and glycolytic processes (5, 6). Glycated hemoglobin (HbA1c) is an important biomarker widely used to assess metabolic health (7, 8). HbA1c is a glycosylated hemoglobin molecule that is produced in red blood cells to reflect average blood sugar levels over the past 2–3 months (9). Consequently, HbA1c, as a stable and objective biomarker, plays a crucial role in the diagnosis and long-term management of diabetes (10).

Glucose metabolism is a complex physiological process influenced by a combination of genetic and environmental factors. Genetic factors determine an individual's basic ability and sensitivity to glucose metabolism, while environmental factors can disrupt the dynamic balance and homeostasis of glucose metabolism (11). Environmental pollutants are one of the most important factors affecting glucose metabolism (12). For instance, heavy metal pollutants such as lead and cadmium have been associated with detrimental effects on pancreatic β-cells, resulting in diminished insulin synthesis and release. This cascade contributes to elevated fasting blood glucose levels and an increased risk of type 2 diabetes mellitus (T2DM) (13). Although numerous studies have explored the detrimental effects of pollutants such as heavy metals, perfluorinated compounds, bisphenols, and phthalates on glucose metabolism and diabetes risk in adults (14–18), research on these effects in adolescents remains limited.

Currently, antimony (Sb) pollution in the environment is becoming increasingly serious, and its harmful effects have attracted increasing amounts of attention (19). The main sources of Sb pollution are anthropogenic, including municipal waste, smelter surroundings, and emissions from the combustion of antimony-containing fuels (19). It is also used in a variety of products and can enter the body through food consumption, drinking water, and inhalation of contaminated air (20). Sb exposure may exert deleterious effects on various bodily organs, including the pancreas, which plays a pivotal role in glucose regulation (21). While some studies have suggested a positive correlation between Sb levels and the risk of T2DM in adults (22, 23), research on the relationship between Sb exposure and glucose metabolism in adolescents is scarce. Adolescents with disorders in blood glucose metabolism may be at higher risk for T2DM and cardiovascular disease in adulthood (24). Therefore, it is crucial to maintain normal blood glucose regulation to promote health. This requires an assessment of the effects of Sb exposure on adolescents, as Sb is a potential disruptor of blood glucose metabolism.

Therefore, we conducted a cross-sectional study using data from the National Health and Nutrition Examination Survey (NHANES) to assess the relationship between urinary Sb exposure and HbA1c levels among adolescents. HbA1c is a potential marker for the diagnosis of diabetes and is important in reflecting long-term blood glucose levels in humans. By examining the correlation between Sb exposure and HbA1c in adolescents, new perspectives are provided to understand how environmental pollutants affect metabolic health in adolescents.

## 2 Methods

### 2.1 Study population

**Figure 1:**
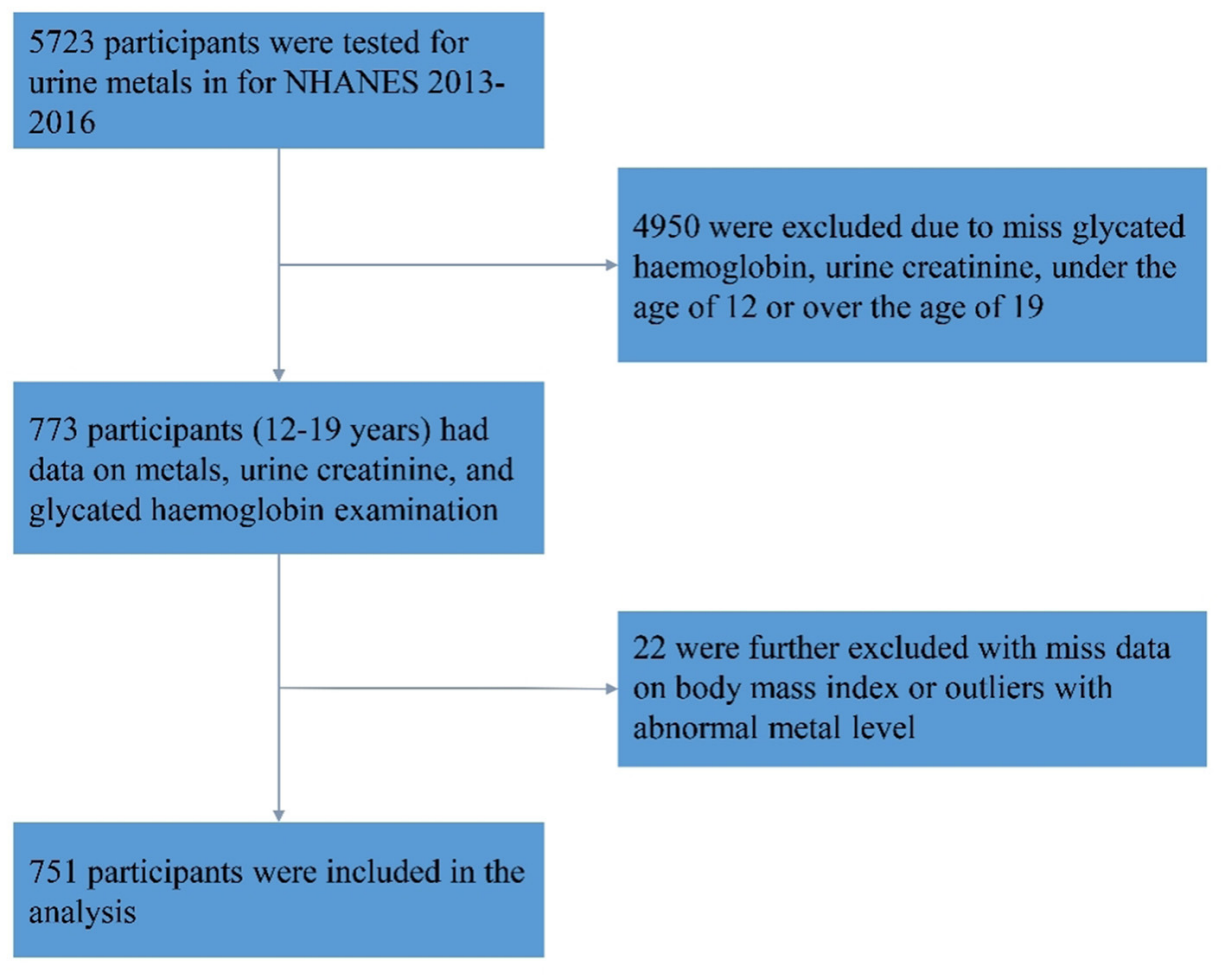
Flow chart of study population exclusion criteria.

The present study utilized data from the NHANES, a program of studies designed to assess the health and nutritional status of adults and children in the United States (25). The NHANES data from the 2013–2014 and 2015–2016 cycles were used in this study. A total of 5,723 participants were tested for Sb in their urine. We excluded 4,950 individuals due to missing HbA1c and Sb data, as well as those who were not within the adolescent age range (aged <12 or >19 years). In addition, after excluding individuals with missing body mass index (BMI) data or abnormal urine Sb levels (defined as 75th percentile plus triple interquartile range), 751 individuals were included in the final analysis (Figure 1).

### 2.2 Measurement of urinary Sb levels in adolescents

Urine samples were collected and processed following established procedures and subsequently sent to the Centers for Disease Control and Prevention's National Center for Environmental Health Laboratory in Atlanta for analysis. Detailed steps for specimen collection, preservation, and processing are available in the NHANES laboratory procedure manual (26, 27). The quantification of metals in urine below the limit of detection (LOD) involves calculating the concentration by dividing the LOD by the square root of 2. Urinary creatinine was used to calibrate the concentration of metals in urine (μg/g cr), a method that ensured the accuracy and consistency of the measurements.

### 2.3 HbA1c in adolescents

During the 2013–2014 and 2015–2016 cycles, NHANES collected whole blood samples from participants aged 12 years or older who underwent physical examinations. The samples were stored at 6°C and subsequently transported to the University of Missouri Columbia for analysis. The percentage of HbA1c in whole blood was measured using the Tosoh G8 Glycohemoglobin Analyzer (Tosoh Bioscience, Inc., South San Francisco, CA.). These measurements were performed using non-porous ion-exchange high-performance liquid chromatography (HPLC) in conjunction with microcomputer technology. The detailed experimental procedures can be found on the NHANES website (28).

### 2.4 Covariates

A range of covariates were considered in the analysis of this study. The variables included age, sex (male and female), race/ethnicity (Mexican American, other Hispanic, non-Hispanic White, non-Hispanic Black, and other races), BMI, family poverty-income ratio (PIR), serum cotinine levels, survey cycles (2013–2014 and 2015–2016), and physical activity (inactive or active). Physical activity was self-reported by the participants and categorized based on their engagement in vigorous or moderate recreational physical activity. Participants were classified as “inactive” if they reported engaging in <10 min of such activity per week, while those reporting more than 10 min per week were classified as “active”. BMI was calculated by dividing the weight in kilograms by the square of the height in meters. For children and adolescents, BMI was compared to the growth charts from the CDC, which show the typical ranges of BMI according to age and sex. Based on these charts, BMI can be classified into four categories: underweight, normal weight, overweight, and obese. Underweight is defined when the BMI falls below the fifth percentile, normal weight when it ranges between the fifth and eighty-fifth percentiles, overweight when between the eighty-fifth and ninety-fifth percentiles, and obese when exceeding the ninety-fifth percentile. Family PIR was categorized into two groups: <1, indicating that the family income was below the federal poverty line, and ≥1, signifying that the family income was equal to or above the federal poverty line. Serum cotinine levels were classified as <1 ng/mL, 1–9.9 ng/mL, or ≥10 ng/ml.

### 2.5 Statistical analyses

The baseline characteristics of the study population were analyzed descriptively. Continuous variables were expressed as the means with standard deviations (Mean ± SD), and categorical variables were presented as percentages. The distribution of urinary Sb levels was characterized using the geometric mean (GM) and percentiles. We conducted a survey-weighted linear regression analysis to assess the impact of urinary Sb concentrations on HbA1c levels. The results were transformed and expressed as a percent change. The percentage differences of HbA1c levels in the blood per doubling of urinary Sb levels were calculated as (e ^(ln2 × β)^ - 1) × 100% (29). Additionally, we created a categorical variable based on the urinary Sb concentrations for further analysis. We applied a linear trend test to explore potential non-linear relationships between Sb and HbA1c across tertiles of urinary Sb, with the first tertile serving as a reference. The percent change in HbA1c associated with tertiles of urinary Sb was calculated as (e ^β^ – 1) × 100% (30, 31). We utilized restricted cubic splines (RCS) to investigate the dose-response relationship between urinary Sb and HbA1c levels (32). Three knots were incorporated into the RCS models, positioned at the 10th, 50th, and 90th percentiles of the natural logarithm-transformed metal concentration. The adjustment variables included age, sex, BMI, race, family PIR, serum cotinine level, physical activity and survey cycle. Furthermore, we conducted sensitivity and subgroup analyses to evaluate the potential influence of supplementary variables on the relationship between urinary Sb concentrations and HbA1c levels in the blood, and to ensure the robustness of our results. We evaluated the relationship between urinary Sb concentrations and HbA1c levels by excluding participants with serum cotinine levels ≥1 ng/mL and those who were overweight/obese. Furthermore, we performed a subgroup analysis stratified by sex.

Statistical analyses were carried out using R software (version 4.3.1), with statistical significance defined as *P* < 0.05 (two-tailed). The “survey” and “rms” packages were used to implement the survey-weighted linear regression analysis and the RCS model, respectively.

## 3 Results

### 3.1 Study population characteristics

The characteristics of the 751 individuals included in this study are detailed in Table 1. Among the participants, the average age was 15.4 ± 2.2 years, 49.8% were male, and 50.2% were female, 26% were non-Hispanic White, 59.8% were underweight or normal weight, 70% were at or above the poverty level, 81.4% had serum cotinine levels <1 ng/mL, and 63.4% were physically inactive.

**Table 1:** Characteristics of included participants from NHANES 2013–2016 (*N* = 751).

**Characteristics**	**Means ±SDs/*N* (%)/Median (25th, 75th)**
Age (years)	15.4 ± 2.2
**Sex**	
Male	374 (49.8)
Female	377 (50.2)
**Race/ethnicity**	
Mexican American	171 (22.8)
Other Hispanic	87 (11.6)
Non-Hispanic White	195 (26.0)
Non-Hispanic Black	173 (23.0)
Other race	125 (16.6)
**BMI (kg/m** ^2^ **)**	
Underweight/Normal weight	449 (59.8)
Overweight	144 (19.2)
Obese	158 (21.0)
**Family PIR**	
<1	225 (30.0)
≥1	526 (70.0)
**Serum cotinine (ng/mL)**	
<1.0	611 (81.4)
1.0–9.9	73 (9.7)
≥10	67 (8.9)
**Survey cycle**	
2013–2014	400 (53.3)
2015–2016	351 (46.7)
**Physical activity**	
Inactive	476 (63.4)
Active	275 (36.6)

NHANES, National health and Nutrition Examination Survey; SD, standard deviation; BMI, body mass index; PIR, Family income to poverty ratio.

### 3.2 Distribution of urinary Sb levels

Table 2 presents the distribution of urinary Sb levels in the adolescents. The LOD for urinary Sb was 0.022 μg/L. The creatinine-corrected Sb level had a geometric mean of 0.049 μg/g creatinine. Moreover, the median urinary Sb concentration was 0.048 μg/g creatinine, with an interquartile range (IQR) of 0.033-0.143 μg/g creatinine.

**Table 2:** Distributions of maternal urinary Sb concentrations (*N* = 751).

**Urinary Sb concentrations**	**LOD (μg/L)**	**GM**	**Percentile**				
			**5th**	**25th**	**50th**	**75th**	**95th**
Uncorrected Sb (μg/L)	0.022	0.056	0.016	0.031	0.057	0.094	0.211
Creatinine-corrected Sb (μg/g creatinine)		0.049	0.020	0.033	0.048	0.069	0.143

Sb, antimony; LOD; limit of detection; GM, geometric mean.

### 3.3 The associations between urinary Sb exposure and HbA1c

The association between urinary Sb exposure and HbA1c levels was investigated, and the results are presented in Table 3. According to the unadjusted model, a doubling of urinary Sb levels was associated with a 1.14% (95% CI: 0.61–1.67) change in HbA1c. After adjusting for potential confounding factors, the positive correlation persisted, with a percentage change of 0.93% (95% CI: 0.42–1.45) in the HbA1c level for every doubling of the urinary Sb concentration.

**Table 3:** Associations between urinary Sb exposure and HbA1c levels.

**Urinary Sb**	**Percentage change (95% CI) in glycated hemoglobin**			
**(mg/g creatinine)**	**Unadjusted model**	***P***	**Adjusted model**	***P***
Per 100% increase	1.14 (0.61, 1.67)	<0.001	0.93 (0.42, 1.45)	0.001
**Tertiles**				
T1 (<0.038)	Reference		Reference	
T2 (0.038–0.059)	0.58 (-0.57, 1.76)	0.307	0.45 (-0.44, 1.35)	0.298
T3 (≥0.059)	2.58 (0.91, 4.28)	0.004	1.45 (0.38, 2.53)	0.012
*P* for trend	0.003		0.011	

Sb, antimony; CI, confidence interval.

Adjusted model adjusted for age, sex, body mass index, race, family income to poverty ratio, serum cotinine level, physical activity and survey cycle.

P for trend across tertiles of urinary Sb.

**Figure 2:**
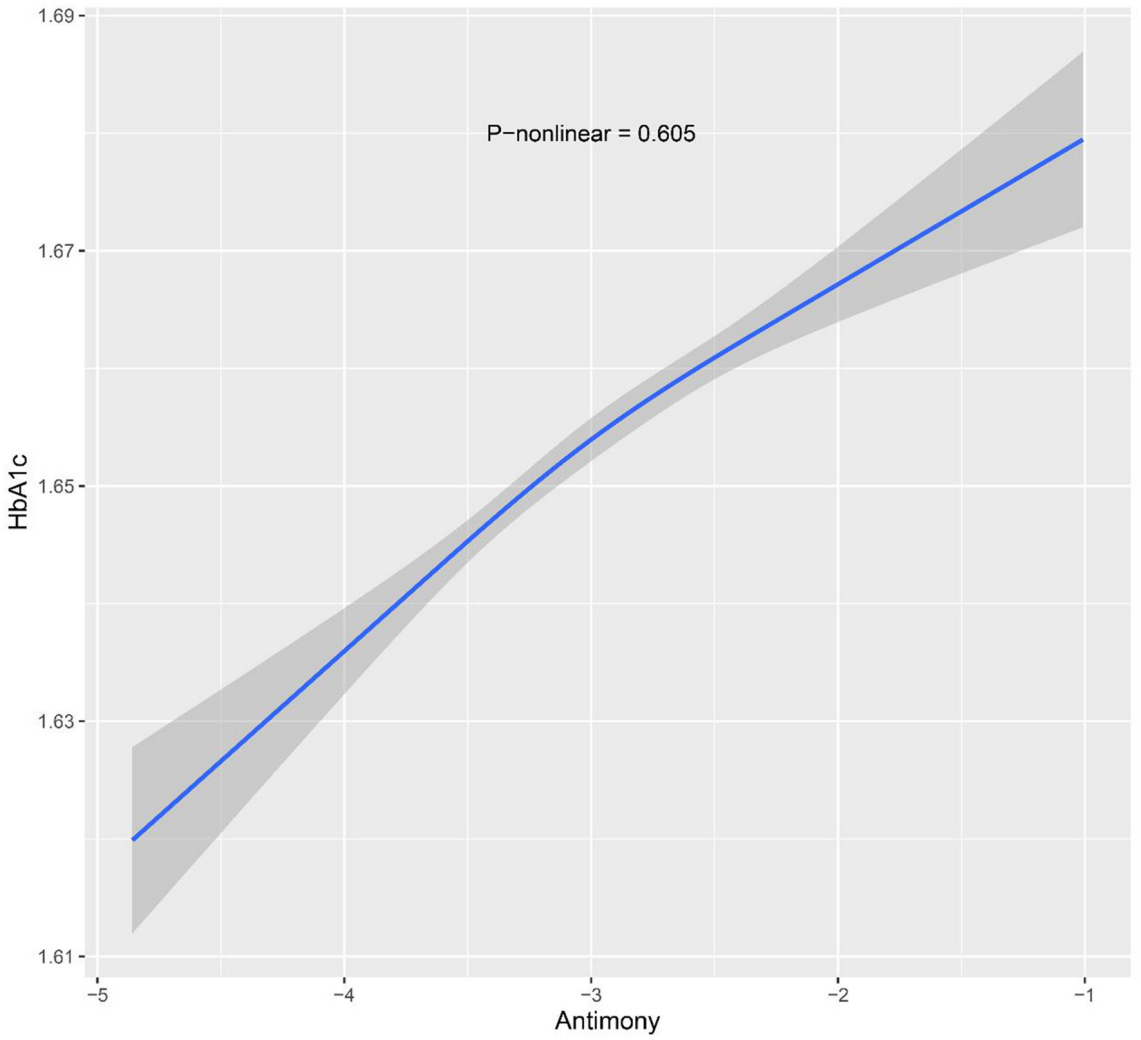
Restricted cubic spline models for ln-transformed Sb and HbA1c. Knots were placed at the 10th, 50th and 90th percentiles. The model was adjusted for age, sex, body mass index, race, family income to poverty ratio, serum cotinine level, physical activity and survey cycle.

Moreover, the participants were stratified into three groups based on their urinary Sb concentrations. According to the adjusted model, HbA1c levels were significantly higher in the group with the highest exposure (T3) compared to the group with the lowest exposure (T1). Specifically, the percent change in HbA1c was 0.45% (95% CI: −0.44, 1.35) in the moderate Sb exposure group (T2) and 1.45% (95% CI: 0.038, 2.53) in the highest Sb exposure group (T3). Although no statistically significant differences were observed in the moderate Sb exposure group (T2), the linear trend test showed a significant linear relationship between urinary Sb concentration and HbA1c (*P* for trend = 0.011). Furthermore, the results of the RCS analysis support a linear relationship between urinary Sb concentration and HbA1c level (*p* = 0.605) (Figure 2).

### 3.4 Sensitivity analyses

According to the sensitivity analysis (Table 4), the relationship between urinary Sb concentration and HbA1c level remained consistent, even after excluding participants with serum cotinine concentration ≥ 1 ng/mL and those who were overweight or obese. The results revealed a significant positive correlation between urinary Sb concentration and HbA1c level, with a percentage change of 1.58% (95% CI: 0.88–2.28) when overweight or obese participants were excluded. In addition, when the study subjects were categorized into different groups according to Sb exposure, we observed a percentage change in HbA1c of 1.36% (95% CI: −0.26, 2.99) and 3.81% (95% CI: 1.59, 6.08) in the moderate Sb exposure group (T2) and the highest Sb exposure group (T3), respectively, when compared with the lowest exposure group (T1). Moreover, the linear trend test revealed a significant linear relationship between urinary Sb concentration and HbA1c (*P* for trend = 0.002). In addition, we performed sensitivity analyses by excluding participants with serum cotinine levels ≥ 1 ng/mL to further investigate the relationship between urinary Sb levels and HbA1c. The analysis showed a significant positive association between urinary Sb exposure and HbA1c levels (Percentage change, 1.14, 95% CI: 0.49–1.80). The percentage changes were 0.36% (95% CI: −1.30, 2.04) and 2.42% (95% CI: 0.57, 4.32) in the moderate Sb exposure group (T2) and the highest Sb exposure group (T3), respectively, compared with the lowest exposure group (T1). The linear trend test showed a significant linear relationship between urinary Sb concentration and HbA1c level *(P* for trend = 0.013).

**Table 4:** Association of urinary Sb exposure and HbA1c levels by excluding the participants who were overweight/obese (Analysis 1) and with serum cotinine ≥ 1 ng/mL (Analysis 2).

**Urinary Sb**	**Percentage change (95% CI) in glycated hemoglobin**			
**(mg/g creatinine)**	**Analysis 1**	***P***	**Analysis 2**	***P***
Per 100% increase	1.58 (0.88, 2.28)	<0.001	1.14 (0.49, 1.80)	0.002
**Tertiles**				
T1 (<0.038)	Reference		Reference	
T2 (0.038-0.059)	1.36 (-0.26, 2.99)	0.095	0.36 (-1.30, 2.04)	0.659
T3 (≥0.059)	3.81 (1.59, 6.08)	0.002	2.42 (0.57, 4.32)	0.013
*P* for trend	0.002		0.013	

Sb, antimony; CI, confidence interval.

Analysis 1 adjusted for age, sex, serum cotinine level, race, family income to poverty ratio, physical activity and survey cycle.

Analysis 2 adjusted for age, sex, body mass index, race, family income to poverty ratio, physical activity and survey cycle.

P for trend across tertiles of urinary Sb.

### 3.5 Subgroup analyses

**Figure 3:**
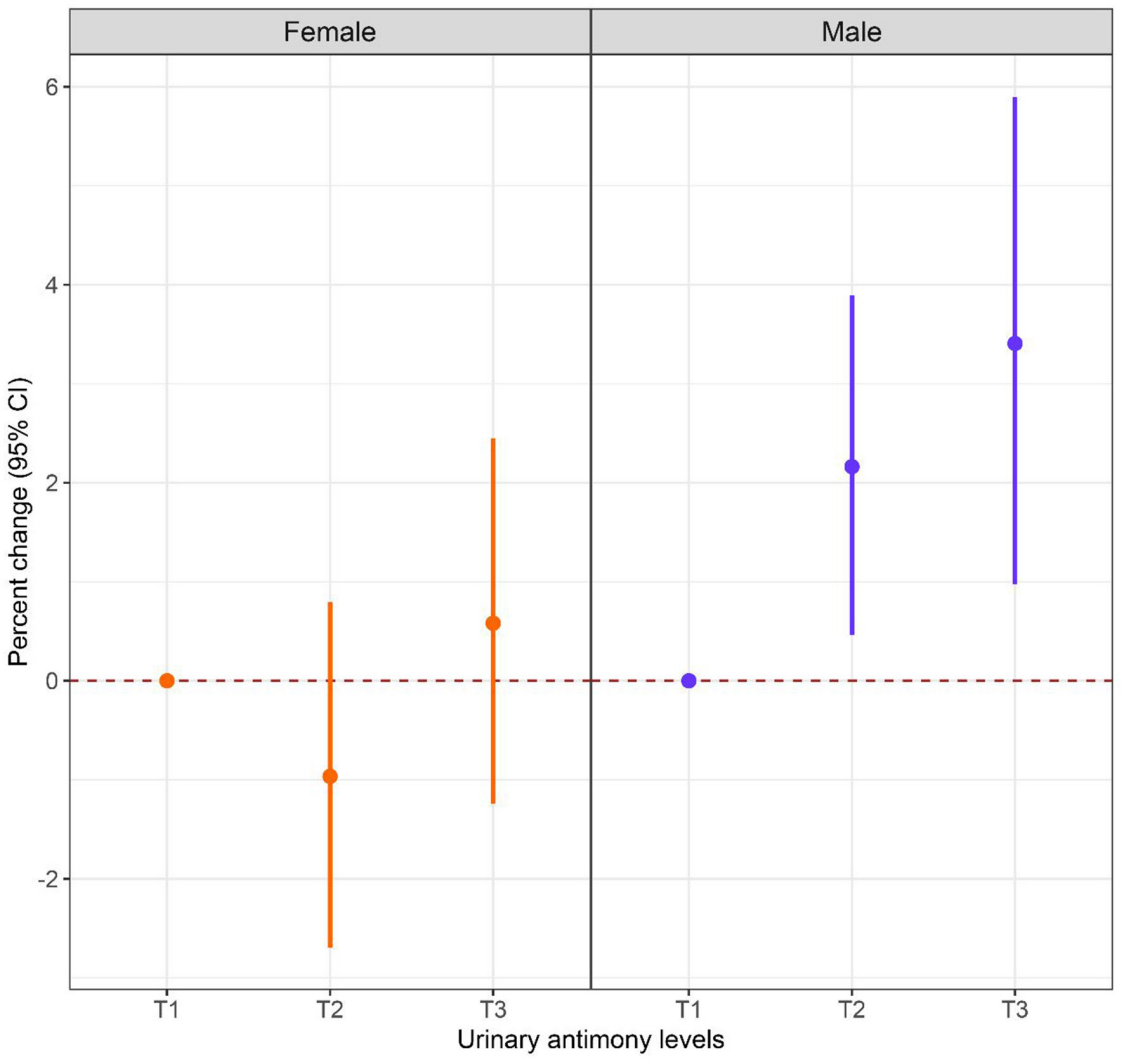
Associations between urinary Sb exposure and HbA1c, stratified by sex. Models were adjusted for age, body mass index, race, family income to poverty ratio, serum cotinine level, physical activity and survey cycle.

**Figure 4:**
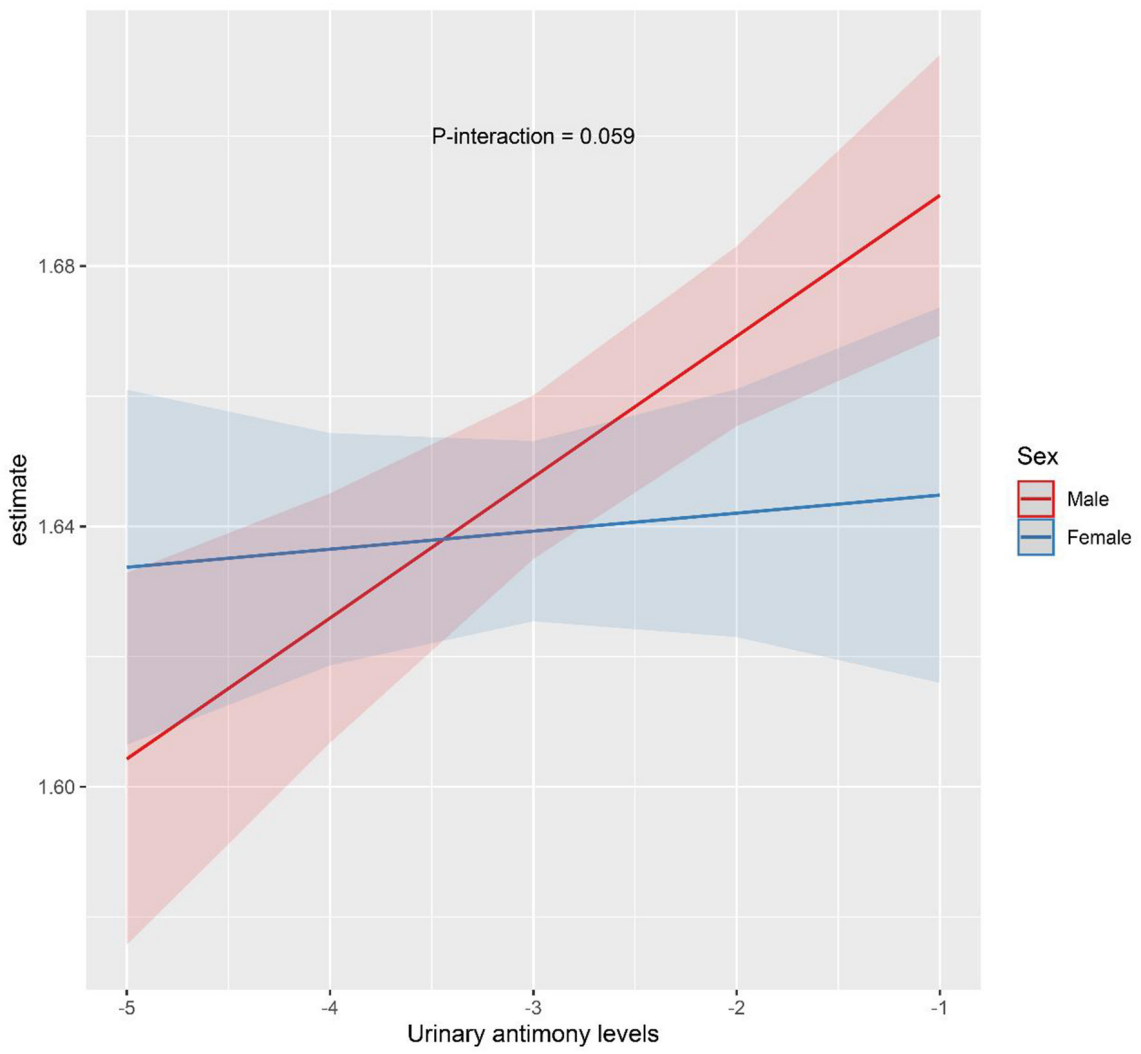
The interaction effect of sex on the association between urinary Sb exposure and HbA1c. Models were adjusted for age, body mass index, race, family income to poverty ratio, serum cotinine level, physical activity and survey cycle.

Sex-stratified analysis were performed to explore the relationship between Sb exposure and HbA1c levels in different gender subgroups, and the results are shown in Figures 3, 4. After adjusting for possible confounders, we found a significant correlation between urinary Sb exposure concentrations and HbA1c levels in male adolescents. HbA1c levels increased by 2.16% (95% CI: 0.46, 3.89) and 3.40% (95% CI: 0.97, 5.89) in the intermediate (T2) and highest (T3) levels of urinary Sb exposure, respectively, compared to the group with the lowest (T1) levels of urinary Sb exposure. In contrast, no significant positive association between urinary Sb exposure and HbA1c was found in female adolescents. And there was no significant interaction between sex and Sb exposure (*P* interaction = 0.059).

## 4 Discussion

The present study evaluated the relationship between urinary Sb exposure and HbA1c levels in adolescents, and to our knowledge, this is the first study to assess this relationship. The results of the present study showed that HbA1c level were increased in adolescents with higher urinary Sb concentrations. These findings suggest a potential association between Sb exposure and altered glucose metabolism in adolescents, which warrants further investigation and consideration in public health strategies. In addition, the study identified a sex-specific effect on the association between urinary Sb exposure and HbA1c levels. While the reasons for this sex-specific effect require additional research, the need for sex-specific analyses in future studies and the importance of considering sex-related factors in environmental health research should be emphasized.

Antimony is a silvery-white heavy metal used in a wide range of industrial applications, including metal alloys, batteries and fireproofing materials (33, 34). Antimony emissions are mainly from anthropogenic sources such as coal combustion, waste incineration and ore mining (34). Additionally, traffic exhaust and brake friction contribute significantly to Sb pollution in urban areas (33). People are exposed to Sb mainly through breathing, eating and drinking (35). After entering the human body, antimony mainly accumulates in the lungs, liver and kidneys, and can also enter red blood cells (33, 36), and is slowly excreted from the body, mainly through urine and feces (36–38). Previous studies have shown that antimony concentrations in urine can be used as an indicator of Sb exposure levels (39).

This study provides a novel exploration of the relationship between antimony exposure and HbA1c levels in adolescents. Although no previous reports have directly explored the relationship between antimony and HbA1c, studies have evaluated the association between antimony and diabetes. For instance, research from both the Tongji Maternal and Child Health Cohort (TMCHC) (40) and the Birth Cohort Study on Prenatal Environments and Offspring Health (PEOH) (39), both of which were conducted in China, revealed a significant association between higher urinary Sb concentrations during pregnancy and an increased risk of gestational diabetes. Furthermore, a cross-sectional study in Wuhan, China found a positive association between urinary Sb concentrations and the risk of diabetes mellitus (41), suggesting a potential link between antimony exposure and diabetes mellitus risk. Similarly, a cross-sectional analysis of the National Health and Nutrition Examination Survey (NHANES, 1999–2010) found that urinary Sb concentrations were positively associated with both the prevalence of diabetes mellitus and insulin resistance (HOMA-IR) (42). Moreover, a previous study suggested that heightened antimony exposure levels may be associated with postprandial increases in blood glucose levels at 1 and 2 h, suggesting a potential link between Sb exposure and the disturbance of glucose homeostasis (39).

However, the mechanisms underlying the association between Sb exposure and HbA1c levels are still unclear. Sb is thought to be an environmental endocrine disruptor that can interfere with normal endocrine function by mimicking the effects of physiologic estrogen. (43, 44). This mimicry has the potential to induce insulin resistance in peripheral tissues and disrupt glucose metabolism, as reported in previous studies (45, 46). Consequently, it is plausible that Sb represents one of the potential mechanisms contributing to elevated HbA1c levels. Furthermore, Sb may also engage in pathogenic mechanisms akin to other metals, encompassing oxidative stress and the potential modulation of insulin gene transcription and expression (45), offering a plausible avenue for understanding its role in contributing to elevated HbA1c levels. Nevertheless, further research is essential to elucidate this link and explore potential avenues for intervention, including broader epidemiologic studies, clinical trials, and molecular mechanistic explorations.

In our study, we observed a sex difference in the relationship between urinary Sb exposure and HbA1c levels. Specifically, we found a significant positive correlation between urinary Sb concentrations and HbA1c levels in males. Currently, the exact mechanisms underlying the relationship between Sb and HbA1c remain unclear, but there are intriguing clues to consider. First, the impact of sex on insulin sensitivity is noteworthy (47, 48). Extensive research has indicated that, under normal blood glucose conditions, females exhibit significantly greater insulin sensitivity than males (47, 49, 50). Estrogens activate the ERα pathway in insulin-sensitive tissues, conferring protection against insulin resistance (51–53). Second, endogenous estrogens are believed to stimulate insulin synthesis and secretion, particularly in females, where they play a protective role in maintaining normal pancreatic β-cell function (47, 48, 54). This finding suggested that females may exhibit greater adaptability when facing factors such as Sb that could potentially affect insulin secretion, thus reducing the risk of rising HbA1c levels. However, these sex differences should be interpreted with caution. Further studies are needed to validate and explore the exact mechanism of the sex-specific association of Sb exposure with HbA1c levels.

This study has several strengths. Firstly, this is the first study to assess the relationship between Sb exposure and HbA1c levels in adolescents, providing a reference for metabolic health assessment in adolescents. Secondly, this study used a nationally representative sample of the U.S. adolescent population and adjusted for many potential confounders, enhancing the reliability of the findings. Finally, the robustness of the results was confirmed through sensitivity and subgroup analyses, accounting for potential variations among the different groups. Nevertheless, several limitations should be acknowledged. First, since this is a cross-sectional study, causality cannot be established, and the study provides correlation rather than causation. Second, although the study proposed hypotheses regarding the connection between Sb exposure and HbA1c levels, it does not offer a specific biological mechanism to elucidate this relationship. In addition, some unmeasured confounding factors (e.g., other environmental contaminants) may impact the results of this study. Future research should aim to address these limitations by employing longitudinal designs to establish causality, exploring the underlying biological mechanisms, and adjusting for additional confounding factors to gain a more comprehensive understanding of the relationship between antimony exposure and glucose metabolism.

## 5 Conclusion

In summary, this study provides preliminary evidence suggesting that Sb exposure may be associated with elevated HbA1c levels in adolescents, with a more pronounced effect in males. However, further research with larger cohorts is necessary to confirm these findings and explore the underlying mechanisms. Early identification of adolescents at increased risk for diabetes due to environmental exposures, such as Sb, may contribute to more effective prevention strategies, particularly by using HbA1c as an early marker for intervention.

## Data Availability

Publicly available datasets were analyzed in this study. The datasets of the National Health and Nutrition Examination Survey are available at https://www.cdc.gov/nchs/nhanes/index.htm.
